# Comorbidities’ Effect on IPF: Pathogenesis and Management

**DOI:** 10.3390/biomedicines13061362

**Published:** 2025-06-01

**Authors:** Andrea Salotti, Maria Chianese, Antonio Romallo, Anna De Nes, Darina Angoni, Alessandra Galantino, Maria Chernovsky, Lucrezia Mondini, Francesco Salton, Paola Confalonieri, Rossella Cifaldi, Pietro Geri, Micol Pividori, Giulia Bandini, Michael Hughes, Marco Confalonieri, Marta Maggisano, Barbara Ruaro

**Affiliations:** 1 Pulmonology Unit, Department of Medical Surgical and Health Sciences, University of Trieste, Hospital of Cattinara, 34149 Trieste, Italy; 2 Department of Experimental and Clinical Medicine, Division of Internal Medicine, Azienda Ospedaliero Universitaria Careggi, University of Florence, 50134 Florence, Italy; 3 Division of Musculoskeletal and Dermatological Sciences, Faculty of Biology, Medicine and Health, The University of Manchester & Salford Royal NHS Foundation Trust, Manchester M6 8HD, UK

**Keywords:** idiopathic pulmonary fibrosis (IPF), comorbidities, pulmonary hypertension, cardiovascular disease, lung cancer, gastroesophageal reflux disease, obstructive sleep apnea

## Abstract

In recent years, there has been a growing recognition within the medical community that idiopathic pulmonary fibrosis (IPF) cannot be effectively managed through a singular focus on the disease itself. Instead, a dual approach is essential, one that not only aims to prolong survival by targeting the underlying pathological mechanisms of IPF but also addresses the numerous comorbidities that frequently complicate the clinical picture for affected individuals. This narrative review seeks to provide a detailed and comprehensive exploration of the various comorbid conditions associated with IPF, which may include cardiovascular disease (CVD), lung cancer (LC), gastroesophageal reflux disease (GERD), obstructive sleep apnea (OSA), and anxiety/depression, among others. By understanding the interplay between these comorbidities and IPF, healthcare providers can better tailor treatment regimens to meet the holistic needs of patients. Furthermore, this review delves into both current management strategies and emerging therapeutic approaches for these comorbidities, emphasizing the importance of interdisciplinary collaboration in clinical practice. By synthesizing the latest research and clinical insights, this review aims to enhance awareness and understanding of the complexities surrounding IPF management, ultimately guiding clinicians in developing more effective, individualized care plans that address not only the fibrotic lung disease but also the broader spectrum of health challenges faced by patients. Through this comprehensive overview, we hope to contribute to the ongoing dialogue about improving quality of life and survival rates for individuals living with IPF.

## 1. Introduction

This decline significantly reduces patients’ quality of life, with acute exacerbations of IPF (AE-IPF) frequently interrupting the disease course. It typically exhibits radiological and histological features consistent with usual interstitial pneumonia (UIP) [1]. Clinically, IPF leads to a gradual decline in lung function, accompanied by worsening respiratory symptoms, such as dyspnea on exertion. This decline significantly reduces patients’ quality of life, with acute exacerbations of IPF (AE-IPF) frequently interrupting the disease course. Ultimately, patients may succumb to respiratory failure or associated comorbidities [2]. The etiology of IPF is multifactorial, encompassing genetic, environmental, immunologic, psychological, and social factors. It predominantly affects older adults, with prevalence estimates ranging from 0.33 to 2.51 per 10,000 individuals in Europe and 2.40 to 2.98 per 10,000 individuals in North America [3].

## 2. Methodological Approach

This narrative review is based on a literature search of PubMed, Embase, and Cochrane Library databases, including articles published up to February 2025. Keywords used included “idiopathic pulmonary fibrosis”, “comorbidities”, “antifibrotic therapy”, “pulmonary hypertension”, “cardiovascular disease”, “lung cancer”, “gastroesophageal reflux disease”, “obstructive sleep apnea”, “anxiety”, “depression”, and specific drug names (pirfenidone and nintedanib). We included systematic reviews, meta-analyses, clinical trials, observational studies, and international clinical practice guidelines relevant to the interplay between IPF, its comorbidities, and management strategies. Articles were selected for their relevance to the epidemiology, pathogenesis, clinical impact, and management of comorbidities in IPF.

Previous guidelines, such as the joint American Thoracic Society (ATS)/European Respiratory Society (ERS)/Japanese Respiratory Society (JRS)/Latin American Thoracic Association (ALAT) guidelines, have addressed the diagnosis and management of IPF [4,5,6]. However, managing the disease remains complex, and prognosis is generally poor. The age-standardized mortality rate for IPF varies significantly, reported to be between 0.5 and 12 per 100,000 persons per year, reflecting regional differences [7].

Current clinical management strategies for IPF include antifibrotic agents (i.e., pirfenidone and nintedanib), anti-inflammatory, and antitussive medications (including oral corticosteroids and opioids) to alleviate cough and enhance quality of life, as well as antacids and proton pump inhibitors (PPIs) to mitigate gastroesophageal reflux. Lung transplantation remains an option, although it is limited to a small subset of patients and is highly dependent on the availability of healthcare resources [1,4,5,6,7]. Without treatment with antifibrotic drugs, IPF can be fatal within 3 to 5 years of diagnosis [8,9,10,11,12,13,14,15].

Furthermore, disease management is significantly complicated by comorbidities, which, in conjunction with exacerbations, further diminish patients’ quality of life, potentially reduce survival, and accelerate disease progression. Increasing evidence underscores the importance of early diagnosis and treatment of comorbidities, which are just as critical as managing IPF itself. Comprehensive evaluations and management of existing comorbidities—including pulmonary hypertension (PH), GERD, OSA, and LC—are essential. Regular evaluations every 3–6 months, or more frequently as needed, are recommended to monitor disease progression. Treatment considerations should encompass both pharmacological (nintedanib and pirfenidone) and non-pharmacological (such as oxygen supplementation and pulmonary rehabilitation) therapies. Optimizing strategies to enhance quality of life should include the treatment of comorbidities, promotion of physical activity, attention to emotional well-being, and the palliation of symptoms [16,17,18,19,20,21,22,23,24].

**Figure 1:**
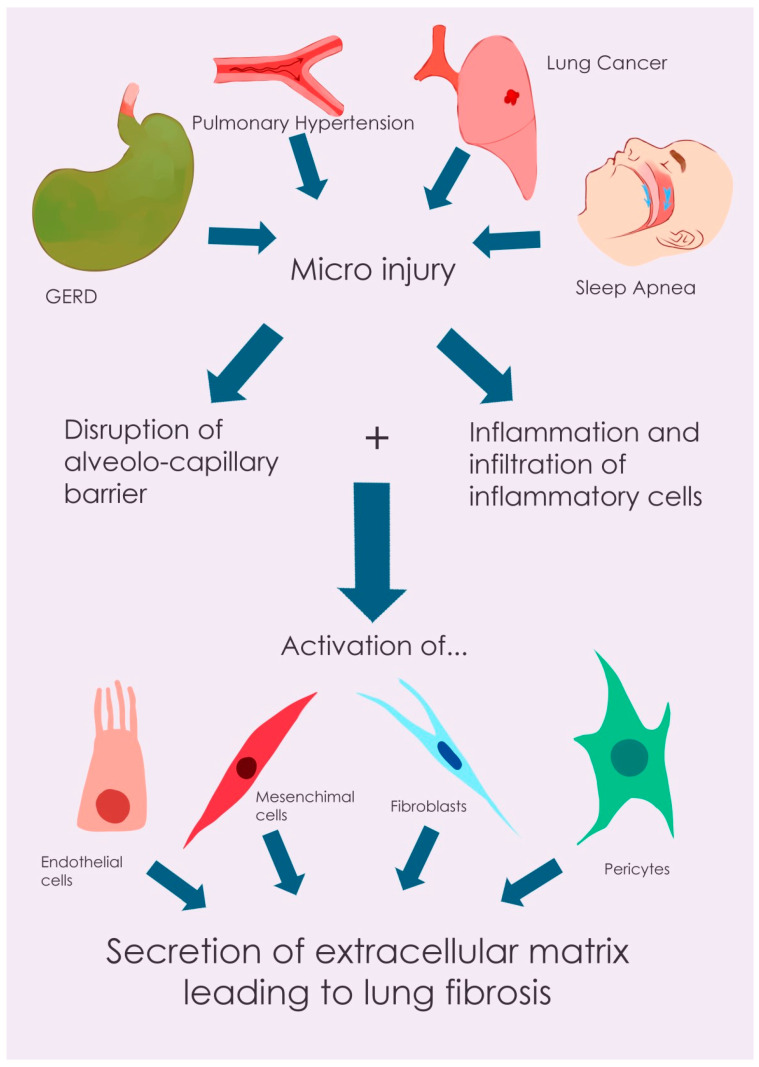
Illustrative diagram of idiopathic pulmonary fibrosis (IPF) pathogenesis, comorbidity interactions, and therapeutic targets. This figure provides a schematic overview of the main pathogenic mechanisms of idiopathic pulmonary fibrosis (IPF), illustrating how prevalent comorbidities (e.g., GERD, IP, and lung cancer) interact with these processes, and highlighting potential therapeutic targets for both antifibrotic treatments and therapies addressing associated conditions.

This literature review aims to assess, verify, and delineate the potential reciprocal interactions between IPF therapies and the identified comorbidities, while also summarizing current management strategies for both IPF and these associated conditions. The diagnosis and management of these comorbidities pose significant challenges for pulmonologists specializing in IPF. Addressing these comorbidities may positively influence the quality of life and survival of IPF patients. The focus of this review is primarily on the impact of comorbidities on IPF, its progression, and its management, including therapeutic strategies for both IPF and its associated conditions (Figure 1).

### Pathogenesis of IPF

The pathogenesis of IPF is characterized by a complex interplay of cellular and molecular events leading to progressive lung fibrosis. The process is initiated by recurrent micro-injuries to the alveolar epithelium, disrupting the alveolar–capillary barrier. This epithelial damage triggers an inflammatory cascade, resulting in the recruitment and infiltration of immune cells, including macrophages. These immune cells release a variety of pro-inflammatory and pro-fibrotic mediators, such as tumor necrosis factor (TNF)-α, transforming growth factor (TGF)-β, monocyte chemoattractant protein-1 (MCP-1/CCL2), macrophage inflammatory protein-1α (MIP-1α/CCL3), and T-helper 2 (Th2) chemokines (CCL17, CCL18, and CCL22). These mediators stimulate the differentiation of various cell types, including fibroblasts, epithelial cells, endothelial cells, fibrocytes, mesenchymal stem cells, and pericytes, into myofibroblasts [25]. Myofibroblasts are the primary effector cells in fibrosis, exhibiting enhanced proliferation, increased extracellular matrix (ECM) production, and contractile properties due to the expression of α-smooth muscle actin (α-SMA) stress fibers. In normal wound healing, myofibroblasts undergo apoptosis after tissue repair. However, in IPF, this process is dysregulated, and myofibroblasts exhibit resistance to apoptosis, leading to their persistent accumulation and excessive ECM deposition [26]. The ECM, under physiological conditions, provides structural support to the lung tissue, maintains mechanical integrity, and contributes to the elastic recoil necessary for proper pulmonary function. Furthermore, it plays a regulatory role in myofibroblast differentiation and activity [27,28]. In IPF, this delicate balance is disrupted, resulting in aberrant lung remodeling characterized by dysregulated ECM deposition, ultimately leading to tissue destruction and functional impairment [28].

## 3. Identified Comorbidities and Their Management

**Figure 2:**
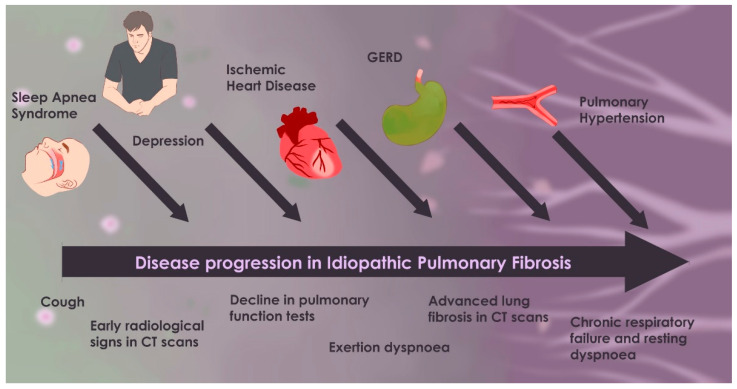
Timeline of IPF progression with comorbidities.

The following comorbidities have been recognized in association with IPF, reflecting their potential impact on disease progression, patient outcomes, and therapeutic strategies (Figure 2). Their management often requires a multidisciplinary approach, integrating recommendations from international guidelines.

Figure 2 illustrates the progression of idiopathic pulmonary fibrosis (IPF) from its early stages—characterized by mild symptoms and limited fibrosis—to its advanced phase, which includes severe dyspnea, extensive fibrosis, and respiratory failure. It also highlights the most significant comorbidities (such as GERD, OSA, pulmonary hypertension, ischemic heart disease, lung cancer, and depression). When these comorbidities manifest, they tend to worsen the disease course, contribute to the overall disease burden, and accelerate clinical decline.

In a 2022 review, Podolanczuk et al. summarized the frequent association of comorbidities which may increase symptom burden and impact survival [29,30,31,32,33], concluding that attention to these and other comorbidities has to be part of a comprehensive evaluation and management of patients with IPF. In particular, in the study by Kreuter et al., the median survival of patients with IPF decreased from 66 months for those without comorbidities to 35 months for those with four to seven comorbidities [32]. Prevalent comorbidities and related complications had previously been examined by Cano-Jiménez E. and colleagues in 2018 [34]. The high prevalence of some comorbidities, such as chronic obstructive pulmonary disease (COPD), LC, and CVD, may coexist with IPF partially due to shared risk factors (smoking, older age, and genetic predisposition), while others may arise as a consequence of IPF itself (e.g., AE-IPF or PH).

### 3.1. Respiratory Comorbidities

#### Pulmonary Emphysema (Combined Pulmonary Fibrosis and Emphysema)

Emphysema and/or COPD have been reported in 6–67% of patients with IPF, and ~30% of patients with IPF have emphysematous changes on imaging [35,36]. The coexistence of these two patterns is typical of combined pulmonary fibrosis and emphysema (CPFE) [37], which is common in male smokers, with the upper lobes being predominantly affected by emphysema and the lower lobes typically fibrotic. It is not clear whether CPFE is associated with a higher or lower mortality risk than IPF alone, but it is certainly higher than in emphysema alone [38].

Patients with CPFE commonly demonstrate paradoxically preserved lung volumes, despite the severity of dyspnea [33], and/or a combination of obstructive and restrictive ventilatory defects, accompanied by substantial diffusion impairment (measured as diffusing capacity of the lungs for carbon monoxide, DLCO) on pulmonary function tests [39]. Prognostically, DLCO values and the extent of emphysema on high-resolution computed tomography (HRCT) are significant. Given a consistent level of fibrosis, emphysema appears to exert an additive negative effect on outcomes [37,38,40].

CPFE is frequently complicated by LC and PH (reported in 15–30% of CPFE patients). Both LC and PH are predictors of mortality in CPFE, along with age and DLCO values [35,41,42,43].

Regarding treatment, pivotal trials for both Pirfenidone (ASCEND) and Nintedanib (INPULSIS) included patients with emphysema, and both antifibrotic drugs seem to have a similar effect in CPFE as in IPF alone [44,45]. In accordance with the ATS/ERS/JRS/ALAT guidelines and research statements [6,39], management should include antifibrotic treatment, bronchodilator therapy (e.g., LABA-LAMA or LABA-LAMA-ICS), smoking cessation, oxygen supplementation for desaturation, pulmonary rehabilitation, and vaccinations [36,37,38,39,46].

### 3.2. Cardiovascular Disorders

#### 3.2.1. Pulmonary Hypertension

Pulmonary hypertension (PH), defined hemodynamically as a mean pulmonary arterial pressure (mPAP) > 20 mmHg at rest confirmed by right heart catheterization (RHC), complicates several ILDs, including IPF, contributing to increased disease burden and poor prognosis [47]. Patients with WHO Group 3 PH (PH due to lung diseases and/or hypoxia) related to pulmonary fibrosis have a worse prognosis compared with other types of PH [48]. The COMPERA registry reported estimated 5-year survival rates of 14% in patients with PH-ILD compared to 51.8% in patients with idiopathic pulmonary arterial hypertension (PAH) [49]. Any degree of PH severity impacts survival, with mortality increasing further with severe PH (Pulmonary Vascular Resistance, PVR >5 Wood Units, especially >8 Wood Units) [50,51].

The prevalence of PH in IPF patients ranges from the range of 8–15% at diagnosis to the range of 35–44% prior to lung transplantation evaluation, and up to 86% during lung transplantation [52]. Comorbidities like emphysema, OSA, cardiac diastolic dysfunction, or pulmonary thromboembolism can exacerbate PH in IPF [46,53,54,55]. Two main phenotypes of PH in IPF exist: PH developing secondary to extensive fibrosis, and a smaller subset (10%) with severe PH despite mild-to-moderate fibrosis [56].

Mechanisms for PH development in ILD include fibrotic vascular ablation, alveolar–septal remodeling, chronic inflammation, aberrant angiogenesis, and potentially genetic factors (e.g., Bone Morphogenetic Protein Receptor type 2 (BMPR2) and TGF-β pathway) [56,57,58,59,60]. These lead to impaired gas diffusion, ventilation/perfusion (V/Q) mismatch, and eventually right heart failure [57,61]. Impaired right ventricular (RV) function can occur even without overt PH [62].

Clinical manifestations are often non-specific. Brain natriuretic peptide (BNP) and N-terminal pro-BNP (NT-proBNP) can indicate RV overload [58]. PH screening is indicated when hypoxemia and DLCO are disproportionately low, or with severe exercise desaturation. The 6-minute walk test (6MWT) distance is often reduced [63]. Cardiopulmonary exercise testing (CPET) can help identify PH, showing reduced peak oxygen consumption (VO_2_) and increased minute ventilation/carbon dioxide production (VE/VCO_2_) slope [64,65].

Imaging clues include a main pulmonary artery to ascending aorta diameter ratio >1 on chest CT, pulmonary artery enlargement, or RV hypertrophy [66,67,68]. Transthoracic echocardiography (TTE) is the primary non-invasive screening tool, estimating RV systolic pressure (RVSP) [67], though its specificity for PH-IPF can be low [69,70,71]. An echocardiographic score can improve identification of severe PH [72]. RHC remains the gold standard for diagnosis [68,72,73,74]. The Ford Score can aid PH screening in ILD [75]. Exclusion of left heart disease is crucial, as post-capillary PH can occur in ~20% of PH-ILD patients [76]. Currently, specific therapy for PH-IPF is limited. The 2022 ESC/ERS guidelines give a Class IIb recommendation for inhaled treprostinil based on the INCREASE study, which showed improved 6MWD and other secondary endpoints in PH-ILD patients (28% IPF) [74,77]. Post hoc analyses suggested potential antifibrotic properties and benefits in less severe hemodynamics [78,79]. TETON studies are ongoing. Parenteral treprostinil showed some benefit in a small study [80]. Inhaled nitric oxide (iNO) showed mixed results in iNO-PF and REBUILD trials [81,82,83]. Sildenafil showed some benefit in secondary endpoints in the STEP-IPF trial but not primary [84,85,86], and combination with nintedanib (INSTAGE) or pirfenidone (SP-IPF) failed to improve PH outcomes [84,85,86,87,88,89]. Other vasodilators, like riociguat and ambrisentan, were either harmful or ineffective [90,91]. Current ESC/ERS guidelines recommend oxygen therapy for hypoxia, treatment of underlying ILD, and referral for lung transplantation for PH in IPF [68,74]. Pulmonary rehabilitation is also important [74].

#### 3.2.2. Ischemic Heart Disease

Patients with IPF have an elevated risk of CVD, with cardiac issues being the second leading cause of death (~10%) [92,93,94,95,96,97]. Coronary artery disease (CAD) is common, with some studies reporting prevalence up to 68% [95]. Shared pathophysiological mechanisms include endothelial damage, chronic inflammation, and fibrosis [95,98,99,100]. Intermittent hypoxemia can also contribute to atherogenesis [101].

Pulmonary fibrosis has been associated with increased CAD incidence compared to non-fibrotic lung diseases [102]. Both IPF and CAD involve excessive fibrosis [103]. Hypoxia can exacerbate angina, and IPF patients may receive suboptimal cardiovascular preventative care [104,105,106,107]. Low forced expiratory volume in 1 s (FEV1) is a risk factor for CAD, and this association appears stronger for IPF-related FEV1 reduction than for COPD-related reduction [107,108,109,110]. Some studies found higher CAD prevalence in IPF lung-transplant candidates compared to emphysema patients, despite lower smoking rates in the IPF group [111,112,113], though other studies in Asian populations showed lower CAD prevalence, possibly due to ethnic differences [98,113,114,115,116]. Hubbard et al. found increased risk of acute coronary syndrome and angina in IPF patients [93]. Nathan et al. [95] found CAD in 65.8% of IPF patients versus 46.1% in COPD patients, with worse outcomes for IPF patients with significant CAD.

Screening for CAD in IPF is important. HRCT scans, routinely used for IPF monitoring, can also assess coronary artery calcium (CAC), a marker for CAD. Bray et al. found the highest CAC scores to be in IPF, followed by COPD and non-smokers [110]. Nathan et al. showed that HRCT had good sensitivity (81%) and specificity (85%) for detecting significant CAD via calcification assessment, suggesting its utility as a screening tool [117].

#### 3.2.3. Arrhythmias

Arrhythmias (AA) occur in 6–19% of IPF cases [30]. Atrial fibrillation (AF) is particularly common, especially in lung-transplant candidates [2]. Contributing factors include hypoxia, altered pulmonary hemodynamics, CAD, and chronic inflammation [2,95,102]. Biomarkers like C-reactive protein and B-type natriuretic peptide may predict AF risk [118]. Hypoxia increases sympathetic activity, and PH can alter hemodynamics around pulmonary veins, predisposing to AF [103].

The most common arrhythmias are AF and Atrial Flutter (AFL). Orrego et al. found IPF to be a risk factor for post-operative arrhythmias after lung transplantation (25.4% incidence; AF, 17.8%) [119]. Shibata et al. found that decreased FEV1% and FVC% were independent risk factors for AF development in IPF and COPD patients [120]. Azadani et al. identified IPF as an independent predictor of AFL post-lung transplant (13% incidence) [121]. Nielsen et al. also found IPF to be a significant predictor for post-operative AF after lung transplantation [122].

### 3.3. Gastrointestinal Comorbidities

#### Gastroesophageal Reflux Disease

Gastroesophageal reflux disease (GERD) is highly prevalent in IPF, potentially affecting 60–80% of patients, often silently [123,124,125,126]. Esophageal pH monitoring is the gold standard for diagnosis [127,128]. In IPF patients with GERD, reflux is often supine, and acid clearance may be slowed despite preserved esophageal function. Severe GERD can lead to microaspiration of gastric fluid, potentially contributing to alveolar damage, IPF progression, and AE-IPF [129]. Elevated pepsin in bronchoalveolar lavage (BAL) fluid during AE-IPF supports this [129]. GERD may be more frequent in asymmetric pulmonary fibrosis [130].

The proposed mechanism involves microaspiration of gastric contents (acid and pepsin), causing subclinical lung injury and fibroproliferation [53,54,124,131,132,133,134,135,136,137]. Detection of gastric molecules in BALF supports this [124,126,135].

The effect of antacid therapy (e.g., PPIs) on IPF progression is controversial. Some observational studies suggested benefits (slower lung function decline, improved survival, and fewer AEs) [137], but post hoc analyses of antifibrotic trials (pirfenidone and nintedanib) showed no significant benefit of antacid therapy over placebo on IPF progression [6,20,124,138,139,140,141]. Current ATS/ERS/JRS/ALAT guidelines conditionally recommend antacid therapy only for IPF patients with symptomatic GERD [6,39,124,131,142]. Anti-reflux surgery is not routinely recommended due to controversial effectiveness but may be considered for selected patients [141]. GERD is a common side effect of pirfenidone [143].

### 3.4. Sleep-Related Breathing Disorders

#### Obstructive Sleep Apnea

Obstructive sleep apnea (OSA) is surprisingly common in IPF, with prevalence estimates ranging from 59% to 88% in various studies [144,145], contrary to earlier beliefs that increased ventilatory drive in IPF might be protective. IPF patients often exhibit abnormal sleep macroarchitecture (reduced slow-wave and rapid eye movement (REM) sleep, increased stage 1 sleep, frequent awakenings, and reduced sleep efficiency) and microarchitecture (more microarousals), impacting quality of life [146].

The Apnea–Hypopnea Index (AHI) has been found to correlate positively with body mass index (BMI) and negatively with FEV1 and FVC [146]. Reduced lung volumes in IPF (decreased total lung capacity, TLC) may decrease upper airway stability, particularly during REM sleep, when functional residual capacity is further reduced, facilitating airway collapse [145]. This is because lower lung volumes reduce the caudal traction on the upper airway, making it more susceptible to collapse. The pathogenic link is complex: OSA might develop due to IPF-related lung restriction, or OSA-related chronic nocturnal intermittent hypoxia could promote GERD or increase oxidative lung stress, potentially exacerbating fibrosis [146,147,148,149]. Animal models show that intermittent hypoxia can worsen bleomycin-induced lung fibrosis [150].

IPF patients often have an altered breathing pattern (rapid, shallow breathing) that persists during sleep [148]. This pattern, driven by increased lung stiffness and vagal receptor activity, is not alleviated during sleep. Nocturnal hypoxemia (SpO_2_ < 90%) is common, especially during REM sleep, due to alveolar hypoventilation, V/Q mismatch, and reduced diffusion capacity [148]. This sleep-related desaturation can be more severe than exercise-induced desaturation and is associated with worse survival and potentially contributes to PH development via mechanisms like increased endothelin-1 [149,151,152].

Early detection of sleep-related breathing disorders (SRBDs) via nocturnal respiratory polygraphy is crucial [146]. Management includes oxygen therapy for REM-related SBD and continuous positive airway pressure (CPAP) for OSA. While long-term oxygen for established respiratory failure in IPF has not shown survival benefits, treating nocturnal hypoxemia might prevent PH development. CPAP for IPF-OSA has been associated with improved quality of life, sleep, daily function, and potentially survival, especially with good adherence [145,146].

Regarding antifibrotic drugs, nintedanib has been linked to improved anxiety/depression and, thus, potentially sleep quality [78], while pirfenidone’s prescribing information notes insomnia in ~10% of cases [44,153,154].

### 3.5. Oncological Comorbidities

#### Lung Cancer

IPF patients have an approximately fivefold increased risk of developing lung cancer (LC), affecting 3–22% of cases [154,155,156,157,158,159]. Shared risk factors (e.g., smoking) and pro-carcinogenic biological pathways contribute [35,154,155]. The risk is even higher in CPFE (up to 12%) and in individuals with interstitial lung abnormalities (ILAs) [155,156,157,158,159,160]. Annual HRCT is recommended for monitoring IPF progression and early LC detection [160].

Pathogenic links include persistent TGF-β activity, which influences cell growth, metastasis, and cancer progression, with pulmonary fibrosis providing a supportive microenvironment [161,162,163,164,165]. Increased fibroblast foci in IPF are linked to more aggressive cancer [161,162,163,164,165]. Programmed cell death-ligand 1 (PD-L1) expression is increased in both IPF and LC. Genetic and epigenetic alterations (somatic mutations, DNA methylation, telomere dysfunction, p53 mutations, FHIT alterations, abnormal microRNA (miRNA) expression, reduced Connexin 43 (CX43) and gap junction intercellular communication (GJIC), Thy-1 promoter hypermethylation, and reactive oxygen species (ROS) overproduction) are implicated in both conditions [155,161,166,167].

Squamous cell carcinoma (SCC) is often reported as more prevalent than adenocarcinoma (AC) in IPF-associated LC, frequently arising in peripheral, fibrotic areas, particularly honeycomb regions [154,168,169,170,171]. Survival is poor, often driven by malignancy [164,170,171,172].

Treatment is challenging. Surgical resection and radiation therapy may be limited by extensive fibrosis and risk of post-operative complications, like AE-IPF (5–15% incidence; ~50% short-term mortality) [170,171,172,173,174]. Tissue-sparing surgery, careful fluid management, and low-tidal-volume ventilation are strategies to improve outcomes [171,174]. Peri-operative pirfenidone may reduce post-operative exacerbation risk [175]. Proton therapy is an emerging radiation option [176]. Many antineoplastic agents, including PD-L1 inhibitors, carry a risk of drug-induced ILD or worsening fibrosis [177,178,179,180]. Carboplatin-based regimens appear relatively safer [180,181,182,183,184,185,186].

Antifibrotic drugs show some promise. Nintedanib, initially approved for non-small-cell lung cancer (NSCLC) with docetaxel [183,187,188,189,190,191,192,193], targets Vascular Endothelial Growth Factor Receptor (VEGFR), Platelet-Derived Growth Factor Receptor (PDGFR), and Fibroblast Growth Factor Receptor (FGFR), showing antitumor effects in preclinical models [194,195]. Pirfenidone may reverse epithelial–mesenchymal transition and induce apoptosis in cancer-associated fibroblasts [163,165,170,181,182]. Observational studies suggest that antifibrotic therapy (pirfenidone or nintedanib) may be associated with lower LC incidence and LC-related mortality in IPF patients [184,185,186,187,188,189,190,191,192,193]. Some case reports suggest that nintedanib monotherapy might inhibit tumor progression in NSCLC with IPF [195,196,197,198,199,200,201,202,203,204]. Combination therapy (e.g., atezolizumab + pirfenidone) is under investigation [193,204,205,206]. However, IPF patients are often excluded from LC trials, thus limiting data [186,187,188,189].

### 3.6. Neuropsychiatric Comorbidities

#### Anxiety and Depression

Psychiatric comorbidities, particularly anxiety and depression, are highly prevalent in patients with IPF, significantly impacting their quality of life, symptom burden, and overall prognosis [24,206]. Studies indicate that depression (estimated prevalence, ~20–50%) and anxiety (estimated prevalence, ~30–60%) are more common in IPF patients compared to the general population and even some other chronic respiratory diseases [24,30,206]. These conditions are often underdiagnosed and undertreated.

The chronic, progressive nature of IPF; and debilitating symptoms like dyspnea and chronic cough, fatigue, social isolation, and uncertainty about the future contribute to psychological distress. Anxiety and depression can exacerbate physical symptoms, creating a vicious cycle: increased anxiety can worsen the perception of dyspnea, leading to activity avoidance, deconditioning, and further functional decline [206].

Furthermore, psychiatric comorbidities can negatively affect treatment adherence (to antifibrotics, oxygen, and pulmonary rehabilitation), engagement in self-management behaviors, and overall coping abilities, ultimately worsening disease outcomes and increasing healthcare utilization [206].

Management requires a holistic, multidisciplinary approach. Screening for anxiety and depression should be a routine part of IPF care. Non-pharmacological interventions are foundational and include pulmonary rehabilitation (which often incorporates psychosocial support and exercise that can improve mood), cognitive behavioral therapy (CBT), mindfulness-based stress reduction, and patient support groups [207]. Pharmacological treatment with antidepressants (e.g., selective serotonin reuptake inhibitors (SSRIs)) or anxiolytics may be considered, carefully weighing potential benefits against side effects and drug interactions, especially in an older population with polypharmacy [207]. Early palliative care interventions can also address psychosocial and spiritual needs, improving quality of life for both patients and caregivers [21,22].

### 3.7. Acute Exacerbations of IPF

IPF is a progressive disease with variable rates of progression [1]. Acute exacerbations of IPF (AE-IPF) are episodes of acute, clinically significant respiratory deterioration characterized by new widespread alveolar abnormality on CT, not fully explained by cardiac failure or fluid overload, typically developing over less than one month [208]. AE-IPF account for approximately 40% of IPF-related deaths [96,209], with an incidence of 4–20% per year [210] and high associated morbidity and mortality (often >50%). Antifibrotic therapies (pirfenidone and nintedanib) may reduce AE-IPF incidence [208,211,212,213,214].

The etiology of AE-IPF is often unknown but may involve occult triggers, like viral infections, an altered respiratory microbiome (e.g., Staphylococcus and Streptococcus), GERD with microaspiration, or procedural stress (e.g., surgery and BAL) [208,215,216,217,218,219,220,221,222,223]. Risk factors for AE-IPF include advanced functional impairment (low FVC, DLCO, PaO_2_, and 6MWT distance), poor baseline oxygenation, younger age, CAD, and high BMI [224]. Chronic comorbidities like PH and CVD are also implicated [209,210,224,225,226,227,228,229,230].

The impact of specific chronic comorbidities on AE-IPF outcomes is an area of active research. A study by Baig and Yoo [230] using a large inpatient database found that chronic kidney disease (CKD) was associated with significantly increased in-hospital mortality among AE-IPF patients, while diabetes was associated with a reduced risk of death. Older age was correlated with higher mortality. The link between CKD and worse AE-IPF outcomes may relate to systemic impacts of AE-IPF exacerbating pre-existing kidney injury [231,232]. The potential protective effect of diabetes is less clear, though some studies suggest lower acute respiratory distress syndrome (ARDS) incidence in diabetics [233,234,235], but the link to ARDS mortality is not established [236,237].

Management of AE-IPF is largely supportive. Corticosteroids are weakly recommended based on low-quality evidence [4]. Mechanical ventilation carries high mortality and is often a bridge to transplant in selected cases [4,209]. Immunomodulatory agents, sometimes combined with antifibrotics, have been explored in small studies, but large-scale controlled data are lacking [219,220,221,222,223,224,225,226,227]. Antifibrotic drugs have shown efficacy in reducing IPF exacerbation frequency, including post-surgically [238,239]. Real-world studies are crucial for understanding antifibrotic effects on comorbidities, as pivotal trials often exclude patients with significant comorbidities [240,241,242,243,244,245,246,247,248,249,250,251]. Both pirfenidone and nintedanib have demonstrated benefits in slowing FVC decline and reducing AE incidence [240,244,245].

#### Shared Decision-Making and Integrated Management

The management of IPF with multiple comorbidities is inherently complex and necessitates a patient-centered approach grounded in shared decision-making (SDM). SDM is a collaborative process where clinicians and patients work together to make healthcare choices, considering the best scientific evidence, as well as the patient’s values, preferences, and goals of care. Given the progressive nature of IPF, the significant impact of comorbidities on quality of life and prognosis, and the potential side effects and burdens of various treatments (for IPF and its comorbidities), SDM is paramount.

**Figure 3:**
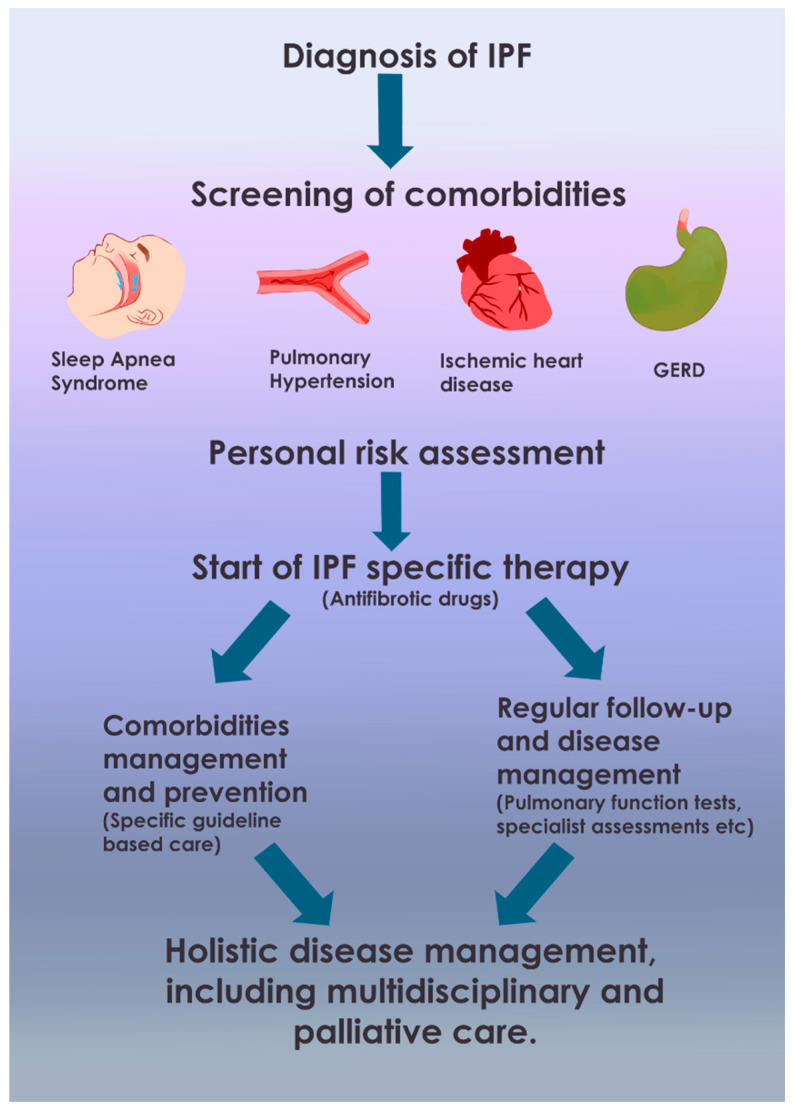
Flow diagram for integrated management of IPF and comorbidities. The flowchart outlines the management of IPF, beginning with diagnosis, followed by comprehensive screening for comorbidities such as cardiovascular issues, GERD, OSA, mood disorders, and cancer risk. An individualized risk assessment guides the initiation of antifibrotic therapy, considering patient-specific factors. Management of comorbidities is conducted through a multidisciplinary approach based on guidelines. Regular monitoring of lung function, symptoms, comorbidities, and treatment side effects is essential, alongside patient education and self-management support. Early involvement of palliative care and continuous shared decision-making ensure personalized and adaptable treatment planning.

Discussions should cover the natural history of IPF, the expected benefits and risks of antifibrotic therapies, the importance of identifying and managing comorbidities, and how these comorbid conditions might influence treatment choices or be affected by IPF therapies. Patient preferences regarding quality versus quantity of life, tolerance for side effects, and personal life circumstances should be actively elicited and incorporated into the care plan. This is particularly relevant when considering interventions for comorbidities that may have their own burdens or when discussing advance care planning and palliative care options. An integrated, multidisciplinary team approach, as discussed below, facilitates effective SDM by providing comprehensive information and support (Figure 3).

## 4. Conclusions and Future Perspectives

Management of IPF extends far beyond targeting pulmonary fibrosis alone; it requires a comprehensive strategy that actively identifies and addresses its numerous and impactful comorbidities (Table 1).

Conditions such as PH, IHD, GERD, OSA, LC, anxiety, and depression are not mere bystanders but can significantly influence IPF progression, symptom burden, quality of life, and survival. Antifibrotic therapies form the cornerstone of IPF management by slowing disease progression, but their optimal use and effectiveness can be modulated by the presence and management of these coexisting conditions (Table 2).

This review underscores the critical need for a multidisciplinary, patient-centered approach. Pulmonologists, cardiologists, gastroenterologists, oncologists, sleep specialists, mental health professionals, palliative care teams, and specialist nurses must collaborate to develop integrated care plans. Routine screening for common comorbidities at diagnosis and during follow-up is essential, guided by international clinical guidelines.

## 5. Future Directions

Despite advancements, significant gaps remain in our understanding and management of comorbidities in IPF. Continuous research is imperative. Future research should focus on the following:

Large-scale, multicenter prospective cohort studies: To better delineate the prevalence, incidence, natural history, and prognostic impact of various comorbidities in diverse IPF populations, and to identify risk factors for their development.

Mechanistic studies: To unravel the shared and distinct pathophysiological pathways linking IPF and its comorbidities (e.g., systemic inflammation, shared genetic predispositions, and impact of hypoxia). This could identify novel therapeutic targets.

Development and validation of personalized therapeutic approaches: Tailoring treatment strategies based on individual patient profiles, including their specific comorbidity burden, genetic markers, and biomarker signatures. This includes investigating the efficacy and safety of antifibrotic agents in IPF patients with specific comorbidities and exploring optimal combination therapies.

Clinical trials for comorbidity management in IPF: Dedicated trials are needed to evaluate the impact of treating specific comorbidities (e.g., aggressive GERD management, CPAP for OSA, and targeted PH therapies) on IPF-related outcomes, including lung function decline, exacerbation rates, and survival.

Implementation and evaluation of multidisciplinary care models: Assessing the effectiveness and cost-effectiveness of integrated, multidisciplinary care pathways in improving patient outcomes, quality of life, and healthcare resource utilization.

Patient-Reported Outcomes (PROs): Greater emphasis on PROs in clinical trials and real-world studies to capture the holistic impact of IPF and its comorbidities, and the benefits of integrated management, from the patient’s perspective.

The analysis of drug–drug interactions between antifibrotic agents and medications used to treat comorbidities in IPF is not within the primary scope of this study; however, recognizing and understanding these interactions may enhance treatment safety and efficacy. Table 2 summarizes the current knowledge on this topic.

In conclusion, effectively managing the complex interplay between IPF and its comorbidities is a cornerstone of modern IPF care. By embracing a holistic, evidence-based, and collaborative approach that prioritizes early detection, guideline-adherent management of comorbidities, and shared decision-making, clinicians can strive to improve not only survival but also the quality of life for individuals living with this devastating disease. The journey requires ongoing research and a commitment to translating new knowledge into better, more personalized patient care.

## Figures and Tables

**Table 1:** Comprehensive summary of common comorbidities associated with IPF.

Comorbidity	Prevalence in IPF	Impact on IPF	Key Tests	Management Strategies	Impact on Antifibrotic Therapy
Pulmonary emphysema and/or COPD	6–67% [35,36].	Mortality > emphysema alone [38]. Additional negative effect on outcomes [37,38,40]. Association with LC and PH in 15–30% of CPFE [35,41,42,43].	PFT DLCO [39], HRCT [37].	Antifibrotics + bronchodilators + smoking cessation + O_2_ supplementation for desaturation + pulmonary rehabilitation + vaccines [6,36,37,38,39,46].	Both antifibrotic drugs have a similar effect on CPFE as on IPF alone [44,45].
Pulmonary hypertension	from 8–15% at diagnosis, up to 86% at lung transplantation [52].	Increased disease burden and poor prognosis [47,48,49,50,51].	TTE estimating RVSP [67], echocardiographic score for severe PH [72]. RHC gold standard [68,73].	O_2_ therapy for hypoxia + antifibrotics + referral for lung transplantation + pulmonary rehabilitation [68,74]. Some evidence for treprostinil [74,77,78,79,80], iNO [81,82,83], and sildenafil [86].	
Ischemic heart disease	Up to 68% [95].	Second leading cause of death (~10%) [92,93,94,95,96,97]. Worse outcomes when significant CAD.	HRCT scans assessing CAC score as a screening tool [117].		
Arrhythmias	6–19% [30].		Biomarkers like CRP and BNP may predict AF risk [118]. decreased FEV1% and FVC% independent risk factors for AF in IPF and COPD [120]. IPF risk factor for arrhythmias after lung transplantation [119,121,122].		
Gastro-esophageal reflux disease	60–80%, [123,124,125,126].	Often silently [123,124,125,126].	Esophageal pH monitoring: gold standard for diagnosis [127,128].	antacid therapy only when symptomatic GERD [6,39,124,131,142]. Anti-reflux surgery not routinely recommended [141].	common side effect of pirfenidone [143].
Obstructive sleep apnea	59–88% [144,145]	Abnormal sleep macro- and microarchitecture impacting QoL [146] with worse survival	Crucial early detection of SRBD via nocturnal respiratory polygraphy [146].	O_2_ therapy for REM-related SBD and CPAP for OSA [145,146].	nintedanib improves anxiety/depression and thus potentially sleep quality [78]. Insomnia: side effect of pirfenidone (~10%) [44,153].
Lung cancer	3–22% [155,156,157,158,159]. Higher risk in CPFE (up to 12%) and in ILAs [38].	Poor survival, often driven by the malignancy [164,170,171]. SCC prevalence > AC, frequently arising in peripheral, fibrotic areas, particularly honeycomb regions [154,168,169,170,171].	Annual HRCT for early LC detection [160].	Surgical resection and radiation therapy limited by extensive fibrosis and risk of post-operative complications [170,173,174]. Tissue-sparing surgery, careful fluid management, and low-tidal-volume ventilation [171,174]. Carboplatin-based regimens are relatively safer than many antineoplastic agents [180]. Combination therapy under investigation [193].	Perioperative pirfenidone may reduce post-operative exacerbation risk [175] Pirfenidone inhibits epithelial–mesenchymal transition and promotes apoptosis in cancer-associated fibroblasts [163,165,170,181,182]. Nintedanib antitumor effects in preclinical models [194,195] and in case reports on NSCLC [202,203,204]. Lower LC incidence and LC-related mortality in observational studies on antifibrotic therapy [184,185,190,191].
Anxiety and depression	Anxiety 30–60% Depression 20–50% >General population and other chronic respiratory diseases [24,30]	Worse quality of life, symptom burden, and overall prognosis [24,206]. Negatively affect treatment adherence, engagement in self-management behaviors, and overall coping abilities [206].	Screening for psychiatric comorbidities should be a routine part of IPF care [207]	Holistic approach: pulmonary rehabilitation, CBT, mindfulness-based stress reduction, and patient support groups [207]. Antidepressants or anxiolytics [207]. Early palliative care interventions, improving QoL [21,22].	

COPD = chronic obstructive pulmonary disease; LC = lung cancer; PH = pulmonary hypertension; CPFE = combined pulmonary fibrosis and emphysema; PFT = pulmonary function test; DLCO = diffusing capacity of the lungs for carbon monoxide; HRCT = high-resolution computed tomography; BNP = brain natriuretic peptide; NTproBNP = N-terminal prohormone of B-type natriuretic peptide; TTE = transthoracic echocardiography; RVSP = right ventricular systolic pressure; RHC= right heart catheterization; iNO = inhaled nitric oxide; CAD = coronary artery disease; CAC-score = coronary artery calcium score; CRP = C-reactive protein; AF = atrial fibrillation; QoL = quality of life; SRBD = sleep-related breathing disorder; CPAP = continuous positive airway pressure; OSA = obstructive sleep apnea; ILAs = interstitial lung abnormalities; SCC = squamous cell carcinoma; AC = adenocarcinoma; CBT = cognitive behavioral therapy.

**Table 2:** Potential drug–drug interactions between antifibrotic agents and medications for comorbidities.

Drug Class	Pirfenidone	Nintedanib
Bronchodilators	No clinically relevant interactions known.	No clinically relevant interactions known.
Inhaled corticosteroids	No significant interaction; no specific management.	No significant interaction; no specific management.
Antiplatelets (e.g., ASA and clopidogrel) [246]	Use with caution: potential increased bleeding risk not documented but theoretically possible.	Increased bleeding risk. Use with caution, especially with ASA or clopidogrel.
Anticoagulants (e.g., warfarin and DOACs) [246,247]	Use with caution. No direct interaction is known, but bleeding risk should be considered.	Increased bleeding risk. Caution advised when co-administered with oral anticoagulants.
Beta-blockers	No significant interaction; no specific management.	No significant interaction; no specific management.
Antiarrhythmics	No significant interaction reported; monitor QT interval if applicable.	No significant interaction reported; monitor QT interval if applicable.
Antacids/PPIs (e.g., omeprazole)	Possible reduced absorption in the presence of elevated gastric pH. Administer separately [248].	No clinically significant interaction observed [249].
Drugs for pulmonary hypertension	No direct interaction with sildenafil [85] or other vasodilators, but additive vasodilatory effects are possible; monitor blood pressure.	No direct interaction is known, but monitor hemodynamic tolerance.
Antidepressants (e.g., SSRIs, SNRIs, and TCAs)	No significant documented interaction.	No clinically significant interaction documented.
Anxiolytics (e.g., benzodiazepines)	No known significant interaction.	No known significant interaction.
Statins	Potential for hepatotoxicity; monitor liver function.	Possible additive hepatotoxicity; monitor liver enzymes
Drugs for lung cancer	Possible interactions with CYP1A2 inhibitors (e.g., some chemotherapeutics). Use caution. (A)	Potential interactions with CYP3A4 (osimertinib) and P-gp substrates. Monitor closely. (B)

(A) Pirfenidone is primarily metabolized by CYP1A2. (B) Nintedanib is a substrate of P-glycoprotein (P-gp) and only marginally involves the CYP system. For both drugs, co-administration with medications that affect hepatic metabolism or coagulation should be monitored carefully.
